# Race and ethnicity moderate the associations between lifetime psilocybin use and crime arrests

**DOI:** 10.3389/fpsyt.2023.1169692

**Published:** 2023-08-24

**Authors:** Grant Jones, Maha Al-Suwaidi, Franchesca Castro-Ramirez, Taylor C. McGuire, Patrick Mair, Matthew K. Nock

**Affiliations:** ^1^Department of Psychology, Harvard University, Cambridge, MA, United States; ^2^Department of Psychology, University of California, Los Angeles, Los Angeles, CA, United States

**Keywords:** race, crime, psilocybin, psychedelics, NSDUH

## Abstract

**Introduction:**

Psilocybin use has been linked to lowered odds of crime-related outcomes across a host of observational studies. No studies have investigated how these associations may differ among those of different races and ethnicities.

**Methods:**

Using a nationally-representative sample of 734,061 adults from the National Survey on Drug Use and Health (2002–2020), we investigated whether race and ethnicity moderate the associations between lifetime psilocybin use and four measures of crime arrests (property crime, assault, serious violence, and miscellaneous crimes).

**Results:**

First, we replicated prior findings and demonstrated that psilocybin confers lowered odds of crime arrests for all four outcomes in question. Second, we demonstrated that race and ethnicity moderate the associations between lifetime psilocybin use and crime arrests for three of our four outcomes. Third, we examined the associations between psilocybin and crime arrests across different races and ethnicities (White, Black, Indigenous, Asian, Multiracial, and Hispanic participants). Psilocybin conferred lowered odds of at least one crime arrest outcome for all racial and ethnic groups except for Black and Hispanic participants.

**Discussion:**

Future investigations should take an intersectional approach to studying the interrelationship of sociodemographic factors, psychedelic use, and crime, examine the structural factors (i.e., systemic racism) that may underlie these results, and investigate whether psychedelics can alleviate mental health disorders that contribute to cycles of recriminalization for communities of color.

## Introduction

Classic psychedelics are naturally-occurring compounds that are known to elicit self-transcendent awe and mystical-type experiences that may have therapeutic effects for a host of mental health conditions (1–3). Accordingly, psychedelic use has been linked to lowered odds of crime arrests (4) and related outcomes such as recidivism (5), intimate partner violence (6), and aggressive behavior (7). Burgeoning research on psychedelic use suggests a promising link between psychedelic use and reduced criminality; however, a clear limitation to this research is the total lack of exploration of how race and ethnicity may impact these associations. Given the overrepresentation of communities of color within the criminal justice system (8), such investigations are imperative for more fully understanding the relationships between psychedelic use and reductions in criminality. Therefore, this study aims to explore how race and ethnicity moderate the associations between psychedelic use and crime arrests.

### Psychedelic use and crime

Several observational studies have linked hallucinogen and psychedelic use to lowered odds of criminal outcomes. Hendricks et al. (5) reported that hallucinogen use predicted lowered odds of recidivism in a longitudinal study of 25,000 adults who were in the criminal justice system, and under community supervision at the time of the study. Furthermore, psychedelic use predicted reduced arrests for intimate partner violence in a sample of 200 incarcerated males with substance misuse problems (6). Similarly, Thiessen et al. (9) conducted a community study and reported that psilocybin and/or LSD use among males was associated with reduced odds of intimate partner violence and greater emotion regulation. Hendricks et al. (10) analyzed data from the National Survey on Drug Use and Health (NSDUH) (2002–2014) and reported reduced odds of criminal behaviors for those with lifetime classic psychedelic use. Recently, Jones and Nock (4) replicated and extended those findings with NSDUH data from 2015 to 2019, and found that lifetime psilocybin use–a classic psychedelic compound that is the active compound within “magic mushrooms”–was associated with reduced odds of multiple crime and arrest variables.

While these studies have provided foundational knowledge on the relationship between psychedelic use and crime, they are not without their limitations. These studies did not conduct any analyses within and between racial groups. Furthermore, while the NSDUH samples were diverse, other samples did not include a representative racial and ethnic composition, which limits the findings’ generalizability. Additionally, while sociodemographic characteristics (i.e., race and ethnicity, income, and educational attainment) may influence psychedelic use and crime rates, these variables were often utilized as covariates rather than tested as potential moderators.

### Race and ethnicity as potential moderators

To date there has been no research to consider the role of race/ethnicity as a moderator in the associations between psychedelic use and crime. Preliminary evidence suggests that race and ethnicity may affect that associations that psychedelic use shares with mental health and behavioral outcomes. Specifically, Jones and Nock (11) and Jones (12) examined whether race and ethnicity moderated the associations between psychedelic use and distress, suicidality, and major depressive episodes (MDEs). These studies found that for White adults, psychedelic use was consistently associated with lowered odds of distress, suicidality, and depression; however, among participants of color, psychedelic use was inconsistently associated with lowered odds of these outcomes. Given this evidence that race and ethnicity affect the associations between psychedelic use and mental health, there is a need for similar investigations into the impact of identity on the associations between psychedelic use and crime outcomes.

### Race and the criminal justice system

Additional research on the role of race and ethnicity in moderating the link between psychedelic use and crime is also important as communities of color are overrepresented in the criminal justice system as a result of racialized laws and policing (13). For example, Black Americans and Native Americans/Alaska Natives are more likely to be arrested for drugs and alcohol than are White Americans, and these disparities are not explained by higher rates of use by these diverse populations (14). Another example is the 18:1 (formerly 100:1) weight ratio for eliciting a mandatory minimum five-year sentence for possession of crack versus powder cocaine, despite these two substances being chemically identical. This policy was created as crack use was associated with the Black community and powder cocaine use with the White community, ultimately leading Black Americans to be incarcerated at vastly higher rates for a functionally identical offense (15–17). These disproportionate arrest rates for substance use crimes are part of America’s broader “War on Drugs”–a policy approach that has tried to use incarceration as the solution to substance use but has consequently led to the mass incarceration of communities of color (18). Additionally, although Asian Americans are reported to be incarcerated at similar or lower rates than White Americans (19), such “model minority” myths have led to a severe dearth of research on Asian American involvement with criminal justice system (19, 20); furthermore, there is suggestive evidence that some subgroups of Asian Americans may have greater involvement with the criminal justice system than do other racial and ethnic groups (21). Hence, given these racialized differences in criminal justice involvement and incarceration, it is important to assess whether such differences might apply to the associations between psychedelics and crime, as a preliminary investigation on this matter can highlight the need to attend to identity and structural factors in future investigations involving these substances and criminality.

### The present study

Thus, the present study is a preliminary inquiry as to whether racial and ethnic identity impacts the associations between psychedelic use and crime using correlational, non-causal data. Specifically, this study seeks to expand on Jones and Nock (4) and test whether race and ethnicity moderates the relationships between lifetime psilocybin use and lowered odds of crime arrests, as this prior work demonstrated associations between lifetime psilocybin use and lowered odds of arrests for various types of crime. In line with Jones and Nock (11) and Jones (12), we hypothesized that there would be fewer and weaker relationships between psychedelic use and crime arrests for participants of color in this sample.

## Positionality statement

Inspired by Smith et al. (22), we wish to state our identities and motivations in order for the reader to better understand the backgrounds of the individuals guiding this inquiry. This paper was drafted by researchers in clinical psychology, and a statistician, who hold Black, Puerto Rican, White, and Arab identities. We are motivated to explore the intersection of race, psychedelics, and crime. Additionally, we are aware that there is clear evidence that the criminal justice system is a core contributor to racial inequality and deleterious outcomes for communities of color within the United States (23–27). This research team recognizes that while there is potential for psychedelics to alleviate conditions that may perpetuate cycles of recriminalization [i.e., racial trauma (28–30), substance use disorders (31, 32), etc.], there is also potential for psychedelics to reify the aforementioned systemic harm if identity factors are not considered when developing psychedelic research paradigms and treatments for individuals involved with the criminal justice system [i.e., the history of exploitative medical treatments and research experiments on communities of color (33, 34) and prison populations (35); history of coercive and exploitative government experimentation with psychedelics (36–38)]. Although our investigation itself is not designed to address such enormous topics in full, we aim for this preliminary inquiry to serve as a gateway for deeper exploration on these topics, as race and ethnicity have remained virtually unexplored in inquiries linking psychedelics to the alleviation of crime.

## Methods

Data for this project are from the National Survey on Drug Use and Health (2002–2020), a yearly survey that collects information on health outcomes and substance use within the United States. The NSDUH is administered in all 50 states and the District of Columbia to a nationally representative sample of U.S. citizens aged 12 years and older. We included adults 18 years and older in the analyses (*N* = 734,061). This study was exempt from review by the Harvard IRB as the data are publicly available.

### Independent variable

Lifetime psilocybin use (yes/no) served as the independent variable, as prior population-based research (4) demonstrated psilocybin use to share a host of protective associations with crime arrests.

### Covariates

The following demographic factors and lifetime use variables served as covariates in our analyses: sex (male or female), age (18–25, 26–34, 35–49, 50 or older), educational attainment (less than high school, some high school or high school graduate, some college or above), self-reported engagement in risky behavior (never, seldom, sometimes, or always), annual household income (less than $20,000, $20,000–$49,999, $50,000–$74,999, $75,000 or more), marital status (married, divorced/separated, widowed, or never married), survey year (2002–2020), and lifetime use of various substances [MDMA/ecstasy, other classic psychedelics (LSD, peyote, and mescaline), other illegal substances (cocaine, heroin, PCP, inhalants) and other commonly misused legal/medicinal substances (pain relievers, tranquilizers, stimulants, sedatives, and marijuana)]. These exact covariates have been used in prior population-based survey studies investigating race, psychedelic use, and deleterious outcomes (11, 12).

Dependent Variables (binary - yes/no): We assessed past year arrests for the following four crime categories: property crime [larceny, burglary, theft], simple assault, serious violence and miscellaneous crimes (including DUI). Data for the “serious violence” variable (which assesses whether one was arrested for aggravated assault, rape, or homicide) exist only from 2008 to 2020.

These variables were selected as Jones and Nock (4) demonstrated that psilocybin shares protective associations with these outcomes. For this study, we combined arrests for three different property crimes into one composite property crime variable, and also combined arrests for DUI with other miscellaneous crimes. The number of outcomes was reduced to four [down from the seven significant outcomes reported in Jones and Nock (4)] to reduce the number of tests within our study and limit the likelihood of a type 1 error (e.g., false positive).

### Putative moderator

In line with Jones and Nock (11) and Jones (12), we decided *a priori* to create and use a three-level race and ethnicity variable [Non-Hispanic White (*n* = 465,081), Non-Hispanic Participant of Color (*n* = 154,113), and Hispanic (*n* = 114,867)] for our moderation analyses that was created from the seven-level race and ethnicity variable included in the publicly available NSDUH dataset. The “Non-Hispanic Participant of Color” category consists of participants from the following racial and ethnic groups: Non-Hispanic Black (*N* = 89,407), Non-Hispanic Native American/Alaska Native (*N* = 10,356), Non-Hispanic Native Hawaiian/Pacific Islander (*N* = 3,627), Non-Hispanic Asian (*N* = 30,081), and Multiracial (*N* = 20,642) individuals. We reduced the number of levels from seven down to three in our moderator in order to reduce the number of interaction tests in our study and optimize statistical parsimony.

Furthermore, a 7-level interaction test may be too stringent a benchmark for a preliminary assessment of the impact of race and ethnicity on psychedelics and crime. As interaction tests entail pooling the variance from White participants and participants of color for every variable in a given model aside from those included in the interaction, one is heavily weighting the model terms of the interaction test around the demographic characteristics and substance use profiles of White participants given that they comprise the majority of our sample. Accordingly, to detect differences between individual participant groups of color and White participants using a 7-level moderator, one needs stark differences within the interaction term or equivalent statistical power between groups, representing inappropriately stringent–and sometimes, impossibly stringent–criteria for a preliminary evaluation of differences such as the one we aimed to conduct in this study.

Thus, we believe using a simplified 3-level moderator retains statistical rigor but provides an appropriately lenient and interpretable preliminary test of differences, and this approach can justify future examinations of the link between psilocybin use and crime using more granular identity measures.

### Analysis

Our analytical plan consisted of three steps, the latter two of which are exactly in line with Jones and Nock (11) and Jones (12). First, we used survey-weighted logistic regression to test whether psilocybin confers lowered odds of crime arrests when examining all available NSDUH data. Although this finding has been demonstrated in two prior studies including many years of NSDUH data (4, 10), we aimed to demonstrate this effect in this study to ensure that the protective associations between psilocybin and crime arrests are present when one examines all available NSDUH data. In addition to the aforementioned covariates, race and ethnicity serve as a control variable for this step of our analyses only. The following steps of our analytical plan consisted of in depth explorations of race and ethnicity, psychedelic use, and crime for which we no longer needed to include race and ethnicity as a covariate.

Next, we used survey-weighted logistic regression to test whether race and ethnicity significantly moderate the associations between psilocybin and our four crime arrest outcomes (property crime, simple assault, serious violence, and miscellaneous crime). As stated above, we used a 3-level moderator for this interaction test: Non-Hispanic White participants (reference group), “Non-Hispanic Participants of Color,” and “Hispanic” participants. Furthermore, while we were aware that our interaction tests would generate beta values, we were not concerned with interpreting the specific beta coefficients yielded by our models as the role of these interaction tests was simply to confirm whether there are significant differences between demographics of color and White participants in the associations that psilocybin use shares with crime arrests. Hence, to evaluate our interaction tests, we simply planned to assess whether our interaction tests yielded significant *p*-values. If either of the two contrasts within our interaction test was significant, we would proceed with the third and final step of our analyses.

In step three of our analyses, we stratified our sample by racial and ethnic identity and examined the associations that psilocybin use shares with crime arrests for each racial and ethnic group. In line with Jones and Nock (11) and Jones (12) we used a six-level racial and ethnic categorization to further examine these associations. The categories were: Non-Hispanic White, Black, Indigenous, Asian, Multiracial, and Hispanic. We combined Native American/Alaska Native and Native Hawaiian/Pacific Islander participants into one “Indigenous” category given the relatively small sample sizes of these two groups, also in line with Jones and Nock (11) and Jones (12).

We decided *a priori* to use this approach–rather than using a six-level or seven-level interaction test for our final assessment of differences–for similar reasons to those we discussed when outlining our putative moderator. As previously stated, when one conducts an interaction test, one pools variance across all participants and fixes all terms in the interaction model–aside from those included in the interaction test–to a singular value. Therefore, this approach means that one is most heavily weighting one’s model terms for all covariates around the backgrounds of White participants, given that they are the most populous group in this sample. However, in this study, we instead aimed to fully reflect the unique substance use and demographic profiles of individual racial and ethnic groups when assessing the relationships between psilocybin and crime. Therefore, in this case, a stratified modeling approach appears more appropriate; with a stratified approach, we could create individual models for the relationships between psilocybin and crime for each racial and ethnic group with covariate terms that reflect the unique backgrounds of each group. Additionally, a stratified approach enhances interpretability as well. When one exponentiates the beta values yielded by such an interaction test (as one would do in order to generate an odds ratio in a standard logistic regression model), one instead generates a “ratio of odds ratios,” a statistic that is difficult to interpret. In sum, a stratified approach is more aligned with the aims of our study and yields results that are easier to understand.

Finally, we did not correct for multiple comparisons in this part of our study; while approaches such as Bonferroni correction can reduce the likelihood of a Type 1 error (false positive), they simultaneously can increase the likelihood of a Type 2 error (false negative) as well. Thus, to balance these two types of potential errors, we instead employ an analytical framework that is similar to those that have been used in foundational population-based survey studies on psychedelics (4, 10–12, 39–43), allowing us to compare any resulting findings to those that currently exist in the research literature on psychedelics.

## Results

Table 1 presents the demographics of our sample, as well as the rates of lifetime psilocybin use, stratified by race and ethnicity. Multiracial individuals reported the highest rates of psilocybin use, whereas Black individuals reported the lowest rates. With the exceptions of Multiracial and Native American/Alaska Native participants, Non-White participants in our sample were less likely than White participants to have used psilocybin in their lifetimes.

**Table 1:** Demographics of our sample, stratified by race and ethnicity.

Characteristic	Non- Hispanic White (weighted %)	Non- Hispanic Black (weighted %)	Non- Hispanic Native American/Alaska Native (weighted %)	Non- Hispanic Native Hawaiian/ Pacific Islander (weighted %)	Non- Hispanic Asian (weighted %)	Non- Hispanic Multiracial (weighted %)	Hispanic (weighted %)	P- value^1^
Lifetime psilocybin use	11.7%	1.7%	10.6%	6.0%	2.7%	14.8%	5.0%	<0.001
Marital status								<0.001
Married	57.4%	33.4%	39.9%	51.9%	62.7%	39.6%	50.0%	
Widowed	6.7%	6.3%	5.9%	2.8%	3.5%	6.6%	3.4%	
Divorced or separated	13.6%	17.7%	19.1%	11.8%	6.5%	17.9%	12.8%	
Never been married	22.3%	42.7%	35.1%	33.5%	27.3%	36.0%	33.8%	
Yearly household income								<0.001
<$20,000	14.0%	31.5%	35.7%	21.2%	13.4%	25.3%	25.4%	
$20,000–$49,999	30.3%	36.5%	37.0%	33.5%	24.3%	33.8%	40.7%	
$50,000–$74,999	18.2%	13.8%	12.7%	16.6%	16.1%	15.7%	14.3%	
$75,000+	37.5%	18.2%	14.6%	28.8%	46.3%	25.3%	19.6%	
Age								<0.001
18–25	12.5%	17.3%	17.2%	18.3%	15.6%	19.6%	19.9%	
26–34	13.9%	17.6%	17.7%	20.3%	20.8%	17.5%	22.2%	
35–49	25.7%	28.3%	27.4%	30.7%	31.6%	22.5%	30.7%	
50+	47.9%	36.8%	37.8%	30.7%	32.0%	40.3%	27.2%	
Educational attainment								<0.001
Less than HS	2.3%	3.5%	5.2%	3.6%	2.3%	3.3%	17.6%	
Some HS/HS grad	36.9%	48.8%	54.6%	45.5%	18.3%	41.3%	44.4%	
Some college or above	60.7%	47.7%	40.2%	50.9%	79.4%	55.4%	38.0%	
Self-reported engagement in risky behavior								<0.001
Never	46.2%	67.9%	55.8%	56.5%	63.5%	46.1%	66.4%	
Seldom	38.6%	22.3%	29.1%	26.8%	26.7%	36.9%	22.7%	
Sometimes	13.9%	8.7%	13.3%	14.6%	8.7%	15.0%	9.4%	
Always	1.3%	1.0%	1.8%	2.0%	1.1%	2.0%	1.5%	
Past year arrest for property crime	0.2%	0.6%	1.2%	0.5%	0.1%	0.4%	0.3%	<0.001
Past year assault	0.3%	0.9%	1.5%	0.3%	0.1%	0.7%	0.4%	<0.001
Past year arrest for serious violence	0.1%	0.5%	0.7%	0.1%	0.0%	0.3%	0.2%	<0.001
Past Year Arrest for Misc. Crime (incl. DUI)	1.1%	2.6%	5.3%	1.5%	0.3%	2.1%	1.9%	<0.001

^1^Chi-squared test with Rao & Scott’s second-order correction.

Table 2 presents the associations between psilocybin and our four crime arrest outcomes for our overall sample. Psilocybin conferred lowered odds of all four outcomes: property crime (aOR: 0.77), simple assault (aOR: 0.80), serious violence (aOR: 0.57), and miscellaneous crimes (aOR: 0.90).

**Table 2:** Associations between lifetime psilocybin use and past year arrests for property crime [larceny, burglary, robbery], simple assault, serious violence, and miscellaneous crime (including DUI).

	Property crime		Simple assault		Serious violence		Miscellaneous crimes (incl. DUI)	
Lifetime use	aOR (95% CI)^1^	*p*-value	aOR (95% CI)	*p*-value	aOR (95% CI)	*p*-value	aOR (95% CI)	*p*-value
Lifetime psilocybin use	0.77** (0.63, 0.93)	0.008	0.80* (0.67, 0.97)	0.020	0.57*** (0.41, 0.79)	9e-04	0.90* (0.83, 0.97)	0.010

^1^*p < 0.05; **p < 0.01; ***p < 0.001; aOR, adjusted odds ratio; CI, confidence interval.

Table 3 presents the results of analyses that test interactions between race and ethnicity and lifetime psilocybin use and the associations of these interactions with past year arrests for property crime, violent crime, and miscellaneous crime (incl. DUI). Our race and ethnicity variable interacted significantly with lifetime psilocybin use, and various individual interactions were associated with our crime arrest variables. Specifically, the interaction between the “Non-Hispanic Participant of Color” category and lifetime psilocybin use was associated with three of our four outcomes: simple assault, serious violence and miscellaneous crime. Furthermore, the interaction between the “Hispanic” category and lifetime psilocybin use was significantly associated with assault. Thus, we proceeded forward with stratifying our sample by race and ethnicity and assessing the relationships that psilocybin use shares with crime arrests for participants of color in the sample. Given that the two moderation tests for property crime were just shy of significance, we also included this variable in our stratified analyses.

**Table 3:** Interactions between race and ethnicity and psilocybin and their associations with past year arrests for property crime [larceny, burglary, robbery], simple assault, serious violence, and miscellaneous crime (including DUI).

	Property crime		Assault		Serious violence		Miscellaneous crimes (incl. DUI)	
Interaction test	Beta (95% CI)^1^	*p*-value	Beta (95% CI)	*p*-value	Beta (95% CI)	*p*-value	Beta (95% CI)	*p*-value
Race/Ethnicity * Lifetime Psilocybin Use								
Non-Hispanic Participant of Color * Lifetime Psilocybin Use	−0.38 (−0.81, 0.04)	0.077	−0.37* (−0.73, −0.01)	0.042	−0.78* (−1.4, −0.15)	0.016	−0.34*** (−0.54, −0.14)	9.38e-04
Hispanic * Lifetime Psilocybin Use	0.32 (−0.01, 0.64)	0.055	0.39* (0.02, 0.76)	0.038	0.43 (−0.18, 1.0)	0.163	−0.06 (−0.26, 0.14)	0.532

^1^*p < 0.05; **p < 0.01; ***p < 0.001; CI, confidence interval.

Table 4 presents the associations between psilocybin use and crime arrests by race and ethnicity. For the White participants, psilocybin conferred lowered odds of arrests for property crime (aOR: 0.78) and serious violence (aOR: 0.58). For Indigenous individuals, psilocybin conferred lowered odds of serious violence (aOR: 0.18) and miscellaneous crimes (aOR: 0.57). For Asian individuals, psilocybin conferred lowered odds of simple assault (aOR: 0.24). For Multiracial individuals, psilocybin conferred lowered odds of property crime (aOR: 0.27), simple assault (aOR: 0.49), and serious violence (aOR: 0.27). For Black and Hispanic participants, psilocybin did not confer lowered odds of any outcomes.

**Table 4:** Associations between psilocybin use and crime arrests, stratified by race and ethnicity (White, Black, Indigenous, Asian, Multiracial, Hispanic).

	Property crime		Simple assault		Serious violence		Miscellaneous crimes (incl. DUI)	
Group	aOR (95% CI)^1^	*p*-value	aOR (95% CI)	*p*-value	aOR (95% CI)	*p*-value	aOR (95% CI)	*p*-value
White	0.78* (0.64, 0.96)	0.019	0.81 (0.64, 1.02)	0.068	0.58* (0.38, 0.88)	0.011	0.91 (0.83, 1.01)	0.069
Black	0.53 (0.27, 1.04)	0.066	0.80 (0.45, 1.43)	0.454	0.45 (0.16, 1.26)	0.127	0.78 (0.54, 1.14)	0.199
Indigenous	0.67 (0.30, 1.49)	0.324	1.66 (0.71, 3.89)	0.242	0.18* (0.05, 0.69)	0.013	0.57* (0.32, 1.00)	0.049
Asian	0.68 (0.19, 2.41)	0.543	0.24* (0.07, 0.85)	0.027	0.78 (0.06, 10.4)	0.848	0.81 (0.31, 2.11)	0.659
Multiracial	0.27* (0.10, 0.74)	0.011	0.49* (0.26, 0.91)	0.024	0.27* (0.08, 0.92)	0.037	0.85 (0.56, 1.30)	0.460
Hispanic	1.02 (0.65, 1.62)	0.919	0.89 (0.50, 1.59)	0.694	0.67 (0.32, 1.41)	0.285	0.97 (0.78, 1.22)	0.815

^1^*p < 0.05; **p < 0.01; ***p < 0.001; aOR, adjusted odds ratio; CI, confidence interval.

## Discussion

The goal of this paper was to provide insight into how race and ethnicity may moderate the relationships between psilocybin use and lowered odds of criminal arrests in a cross-sectional study. We found that a composite “Non-Hispanic Participant of Color” group identifier–consisting of Asian, Black, Native Hawaiian/Pacific Islander, Native American/Alaska Native, and Multiracial participants–was a significant moderator for the relationship between lifetime psilocybin use and lowered odds of past year arrests for three of our four outcomes (simple assault, serious violence, and miscellaneous crimes). We also found that the “Hispanic” identifier was a statistically significant moderator for the relationship between lifetime psilocybin use and lower odds of arrest for assault in the past year– indicating that the aforementioned association differs for Hispanic participants relative to Non-Hispanic White participants.

In examining associations by racial and ethnic identity, we found that all racial and ethnic groups besides Black and Hispanic identities shared at least one protective association between psilocybin and past year crime arrests. While these findings stand in contrast to our hypotheses for this study as well as the findings in Jones and Nock (11) and Jones (12)–which found fewer and weaker protective associations between psychedelic use and deleterious outcomes for all participants of color–these findings may nevertheless be important. Black and Hispanic Americans comprise the majority of individuals who are involved in the criminal justice system (44–47), meaning that our findings mirror the disproportionate arrest rates within these communities. Furthermore, although these findings cannot be used to determine causality, they nevertheless raise important questions about the ways that psilocybin use intersects with race and identity to impact the likelihood of a criminal arrest.

### Potential explanations

There are three potential factors that may help to further contextualize our findings related to psilocybin use, race, ethnicity, and crime. It is also important to note that due to the correlational and non-causal nature of our study, these potential explanations remain speculative and our results should be interpreted with caution. The speculative nature of our explanations is further driven by lack of research into the intersection between psychedelics, race, and crime. Future research is needed to further elucidate our results and clarify whether any of the below explanations indeed underlie our findings.

#### Psilocybin, mental health, and criminalization

First, psilocybin may be linked to the alleviation of mental health disorders that make it more likely for one to interact with the criminal justice system; however, systemic racism toward Black and Hispanic Americans may attenuate this link in a few key ways.

Mental health disorders including trauma-related psychopathology (e.g., PTSD), substance use disorders, and depression have been linked to increased risk of incarceration (48–51). Furthermore, there is clear evidence that incarceration, rather than community-based mental health support, is a prevailing American policy response to mental health issues (52–57) and contributes strongly to this link. Psychedelics like psilocybin have demonstrated preliminary efficacy at treating some of the aforementioned mental health conditions that increase one’s risk of incarceration (31, 32, 58–62), and the potentially salutary effects of naturalistic psilocybin use may underlie the protective associations we have observed in this study.

However, structural racism–particularly racism toward Black and Hispanic individuals–may have attenuated these associations in our study for Black and Hispanic participants. Evidence suggests that Black and Hispanic individuals are incarcerated at disproportionately higher rates when experiencing mental health challenges (63–69). Furthermore, these disparities are often on public display; for instance, one can examine the differences in public response to the crack epidemic of the 1980s versus the current opioid crisis as one clear example (70, 71). As this epidemic of crack addiction predominantly affected the Black community, the overwhelming public response was to stigmatize and incarcerate individuals who were addicted to crack. In contrast, the response to the current crisis of opioid addiction–which has largely impacted White Americans–has been to support individuals who are addicted to opioids by providing increased access to public health services. Therefore, such disparities may have eliminated protective associations between psilocybin and crime arrests for Black and Hispanic individuals in this study. Future research should further examine disparities in public responses to mental illness for Black and Hispanic individuals, and assess how these disparities may impact the relationships between psilocybin use and lowered odds of crime arrests.

Furthermore, racism may also impact the potentially salutary nature of the psychedelic experience. The mindset of the individual consuming a psychedelic substance, as well as the setting in which they consume psychedelics, are reported to have a large impact on the nature of the psychedelic experience (72). This phenomenon is referred to as “set and setting.” Anti-Black and anti-Hispanic racism are widespread in the American “setting” (73, 74) and create disproportionate risk of incarceration for Black and Hispanic individuals who consume illegal substances like psychedelics (68, 75). Downstream, this fact may create more negative mind “sets” for Black and Hispanic individuals who engage in psychedelic use, potentially giving rise to aversive or non-salutary psychedelic experiences and ultimately eliminating protective associations between psilocybin and crime arrests for these groups in this study. Future research should further explore how racism impacts psychedelic experiences and outcomes for Black and Hispanic individuals.

#### Demographic differences in rates of and motivations for psychedelic use

Second, different racial and ethnic groups may use psilocybin at different rates and for differing reasons, potentially contributing to the differences in the associations between psilocybin use and crime arrests in this study. Previous work has demonstrated that people of color in the United States are less likely to use psilocybin relative to White adults (76); further, our current study largely confirms these findings, as we observed here that all participants of color aside from Multiracial individuals reported less psilocybin use relative to White participants. Additionally, the number of protective associations by race and ethnicity in our study seemed related to rates of psilocybin use within specific groups. For instance, Multiracial individuals reported the highest rates of psilocybin use, and accordingly had the highest number of protective associations between psilocybin use and crime arrests in this study. In tandem, Asian participants and Black participants had the lowest rates of psilocybin use; accordingly, there was only one protective association for Asian participants and none for Black participants. Accordingly, these differing rates of psilocybin use may relate to differing motivations for use within different communities. For instance, Indigenous communities–who featured high rates of psychedelic use in our study–are known to have used naturally-occurring psychedelics like psilocybin for worship and healing rituals for thousands of years (77). In contrast, individuals from other racial and ethnic groups may use psilocybin for different purposes, contributing to differences in the associations between psilocybin use and crime arrests. Future research should aim to better understand rates of and motivations for psilocybin use within communities of color, as such investigations can shed further light on our findings.

#### Intersectional identity factors and arrest rates

Third, associations between psilocybin and crime arrests for racial and ethnic minorities may also vary based on intersectional identity factors (i.e., geography, skin tone, etc.). Hence, it is also possible that there are demographic subgroups of participants of color who use psilocybin and are targeted by the police at lower rates, creating associations between psilocybin use and lowered odds of specific types of crime arrests. For example, Indigenous people have been found to be disproportionately targeted in drug-related arrests by law enforcement when they are closer in proximity to the borders of tribal nations (14); thus, Indigenous individuals farther away from such borders may be arrested at lower rates for substance-related crimes than those who are closer. As another example, a 2016 study found that the disproportionate rate of drug arrests for Black Americans is lower when Black people live in areas with fewer White people (78); a 2020 follow-up study found that lower rates of White women in particular were associated with lower disproportionate rates of arrests for Black people (79). Hence, racialized policing may intersect with various sociodemographic identity factors to explain the differing odds of arrest for participants who use psilocybin. Future analyses that explore the interrelationship of policing, intersectional identity, and naturalistic psilocybin use can shed further light on our observed findings.

### Limitations

The current study features several limitations. The current study has a cross-sectional design, which results in not being able to make any causal conclusions. Second, although differing rates and motivations for psychedelic use in different racial and ethnic groups may underlie our results, we do not have data on motivations for psychedelic use to further assess this hypothesis. Thus, future surveys and qualitative studies can collect data on the reasons why various communities of color use psychedelics, as such inquiries can shed light on our findings.

Next, given that our main independent variable was lifetime psilocybin use, we could not control for frequency or recency of psychedelic use within our study. However, given that psychedelic use can promote significant and lasting improvements in deleterious outcomes with just a few administrations (80), we do not believe this limitation significantly hinders our observed findings. Furthermore, the intersection between race/ethnicity and other sociodemographic factors was not included in our analyses, which limits our understanding of how characteristics such as socioeconomic status and educational attainment may serve to further moderate the relationships between race and ethnicity, psilocybin use and crime arrests.

Finally, the broad racial and ethnic categories including within the publicly available NSDUH data and within our study represent a clear limitation to the current work. This limitation is particularly relevant for the “Hispanic” category, as this label is an artifact of colonialism that does not accurately reflect the racial and ethnic diversity of people from Latin American countries. For example, a Pew Research Center survey from 2020 found that Hispanic adults were more likely than White or Black adults to report that the ethnicity questions from the 2020 census did not reflect their identity well (81). In fact, a large majority of Hispanic individuals (81% on average) marked just the Hispanic box and no other race category and two-thirds considered Hispanic as their racial background (82), even though there is much variation in the racial backgrounds and skin-tones of individuals from Latin American countries (83).

### Future directions

There are a number of future directions that are warranted in light of this study. First, future studies can take a mixed-methods longitudinal approach to investigate whether there are causal links between psychedelic use, mental health, systemic racism, and cycles of recriminalization for communities of color. Additionally, these longitudinal inquiries can explore specific domains within this research area such as the link between psilocybin use and arrests for substance use crimes. For instance, the United States is currently experiencing an increase in methamphetamine use, and the arrest rate for this substance has increased by 59% from 2015 to 2019 (84). Thus, future studies can explore specific questions within this research field–such as the relationship between psilocybin, race, and arrest rates for methamphetamine–as well as broad questions related to the link between psychedelics, identity, and crime.

Second, future studies may benefit from approaches that allow for qualitative self-identification of race/ethnicity as these may allow for more accurate reports of identity. Additional demographic questions may also query self-reported race, identity centrality (i.e., “How important is your racial identity to you?”), and perceived social identity (i.e., “How much do I identify as [race/ethnicity]?” and, “How would others that do not know me categorize me?”), as these factors may also mediate our observed findings.

Third, follow-up studies can examine new laws and policies regarding the decriminalization/legalization of psilocybin use in the United States. Currently, psilocybin is designated by the Drug Enforcement Administration (DEA) to be a Schedule 1 substance (no medical potential and a high risk for abuse), greatly limiting scientific inquiry into this substance. Future policy investigations can lead to new laws that make it easier to study the intersection of psilocybin and crime. Furthermore, legislative measures can encourage and support research on psilocybin, which can include allocating funds from state and federal budgets for psilocybin research and forming psychedelic research advisory councils comprised of researchers from diverse backgrounds. Fourth, future works should further examine drivers of psychedelic use in communities of color, as this research domain is generally understudied; relatedly, future investigations should also explore sociodemographic intersectionality as it relates to psychedelics, race, structural inequality, and crime.

Fifth, future studies should also further investigate the results of our interaction tests indicating differences in the associations between psilocybin and crime that exist as the broader group identity level (White, Non-Hispanic Participant of Color, Hispanic). As previously mentioned in our Methods, we did not interpret the beta values yielded by our interaction models as these tests were simply to assess whether further investigation of psilocybin, race, and crime would be warranted for specific racial and ethnic groups. However, these significant interaction tests indicate that there are also differences at the broader group level that may warrant further investigation and understanding. While these significant interaction tests may be driven by any of the aforementioned explanations for our findings, (i.e., the link between psilocybin, mental health, and criminalization; demographic differences in rates of/motivations for psychedelic use; intersectional identity factors and arrests rates), they may also be explained by currently unknown factors as well. Thus, these future investigations can deepen our understanding of the relationship between psychedelics and crime, as well as explain how broader identity factors affect this relationship.

Finally, future psychedelic studies should focus on the inclusion of psychedelic researchers of color, as there are very few funded scholars in the psychedelic research field conducting inquiries on race and psychedelics. This fact likely contributes to the lack of representation of participants of color in existing psychedelic treatment studies (85–87). Further recruitment, retention, and promotion of psychedelic researchers who are from communities of color and underrepresented backgrounds can support this future direction.

## Conclusion

The goal of the current study was to examine the potentially moderating role of race and ethnicity for the associations between lifetime psilocybin use and lowered odds of crime arrests. We found that racial and ethnic identity significantly moderated the associations between lifetime psilocybin use and various outcomes assessing past year arrests. Our findings may be driven by the impact of structural racism on psilocybin use and mental health outcomes in communities of color, differing rates of and motivations for psychedelic use across different racial and ethnic groups, and the impact of intersectional identity on psilocybin use and arrest rates. Future studies should consider more nuanced racial and ethnic frameworks when studying psychedelic use and crime, explore additional sociodemographic and structural factors as potential moderators of the aforementioned associations, and examine whether psychedelics can alleviate mental health disorders that may increase the involvement of communities of color with the criminal justice system. Our findings are correlational and not causal, and thus future studies should also examine potential causal factors that may underlie these results. Overall, this study represents a small step toward better understanding the impact of race, ethnicity, and sociodemographic identity on psychedelic use and crime.

## Data availability statement

Data for this project are publicly available at the following web address: https://www.samhsa.gov/data/data-we-collect/nsduh-national-survey-drug-use-and-health.

## Ethics statement

Ethical review and approval was not required for the study on human participants in accordance with the local legislation and institutional requirements. Written informed consent for participation was not required for this study in accordance with the national legislation and the institutional requirements.

## Author contributions

GJ conceptualized the study, conducted all analyses, and drafted the manuscript. TM, MA-S, and FC-R drafted the manuscript. PM provided statistical guidance. MN provided supervision and edits. All authors contributed to the article and approved the submitted version.

## Funding

GJ received funding support for this article from the “atai Fellowship Fund in Psychedelic Neuroscience”.

## Conflict of interest

The authors declare that the research was conducted in the absence of any commercial or financial relationships that could be construed as a potential conflict of interest.

## Publisher’s note

All claims expressed in this article are solely those of the authors and do not necessarily represent those of their affiliated organizations, or those of the publisher, the editors and the reviewers. Any product that may be evaluated in this article, or claim that may be made by its manufacturer, is not guaranteed or endorsed by the publisher.
